# Nursing Section

**Published:** 1904-02-27

**Authors:** 


### The Hospital.

### Nursing Section.

Contributions for this Section of "The Hospital" should be addressed to the Editor, "The Hospital" Nursing Section, 28 & 29 Southampton Street, Strand, London, W.C.

No. 909.—Vol. XXXV. *SATURDAY, FEBRUARY* 27, 1904.

### Notes on News from the Nursing World.

### THE KING'S INSPECTION OF OSBORNE HOUSE.

The King paid a visit to Osborne House on Saturday, arriving there about 2.15 P.M. He was received by the house governor and the matron, also Sir Schomberg McDonnell, Lord Windsor, and the heads of the departments of the Office of Works. His Majesty inspected every room in the convalescent block, and appeared to be delighted with all he saw, especially the new white furniture. The sisters' duty-room on each floor was also most interesting to the King. His attention was directed to the little cooking range, the gas rings, the tiled cupboard and the medicine cupboard. He next visited the sisters' quarters, which are situated on the first floor in the Durbar wing, where accommodation is provided for four sisters and two extra nurses; also a large sitting room for the sisters, with bath-rooms and lavatories, as well as the matron's bedroom. The matron's private sitting-room, office, and the dining-room for the nursing staff are all on the ground floor of the convalescent block. On leaving the building the King expressed his warm approval of all the arrangements. Osborne House, judged by the character of some of its equipments, might be regarded as a modern surgical hospital.

### QUEEN ALEXANDRA'S MILITARY NURSING SERVICE.

The following changes of station have been made in Queen Alexandra's Imperial Nursing Service :—Miss G. E. Saunder, matron on the s.s. *Plassy* for Indian troopship duty; Miss G. A. Magill and Miss J. G. Powell, sisters to Woolwich and the Royal Arsenal, Woolwich, respectively; Miss E. M. Bickerdike and Miss A. F. Byers, staff nurses to s.s. *Plassy* for Indian troopship duty and the Royal Arsenal, Woolwich, respectively. We are also informed that Miss F. M. Keeble has been appointed nurse at the Hospital for Soldiers' Wives and Children, at Portsmouth.

### THE CASE OF "RAGGING" AT AN ASYLUM.

As the result of the inquiries we have instituted respecting the alleged dismissal of eight nurses, or female attendants, at Tooting Bec Asylum for Imbeciles, we are able to give the official statement of the case, which differs considerably from the rumours which had obtained currency. It appears that one day the week before last, a junior nurse preferred a request to the medical superintendent, in the presence of the matron, that she might be transferred to another ward, giving as the reason that the charge and deputy charge nurses in her ward were "making things too hot for her." Accordingly, the medical superintendent sent for the two nurses, and, in the presence of the matron, told them that any attempt to bully their juniors would be severely dealt with. Forty-eight hours afterwards eight of the nurses repaired to the bedroom of the junior, dragged her out of bed, carried her along to the bath-room, which was close at hand, and endeavoured to give her a cold ducking. The girl resisted so effectually that she was not immersed, only much sprinkled with water, and her lip cut in the fray. Next morning the victim went to the medical superintendent and told him her story, but, when asked for names, she said that she could not give them. A little later the medical superintendent sent for three nurses, whom he suspected, accusing them of the escapade as if he knew that they were the culprits. Two of them admitted the truth, were at once suspended, and instructed to leave the building before noon. To the mother of one, who lives in the country, he telegraphed to expect her daughter that day; the other went to her home in London. As the two delinquents refused to mention the names of the others, no further steps were taken, until six nurses came on their own initiative and declared their complicity in such a manner as to leave the superintendent no alternative but to suspend them also. At a meeting of the Committee, on Thursday, the matter was carefully investigated, the action of the medical superintendent was approved, and the eight nurses were formally dismissed.

### THE INFIRMARY NURSE AND THE "USEFUL INMATE."

At the Central Poor-law Conference held at the Guildhall on Tuesday and Wednesday, the President, Sir John T. Dillwyn Llewelyn, made a passing allusion to nursing in the infirmaries in his opening address. Mr. Harris P. Cleaver, Clerk to the West Derby Union, in a paper dealing with classification, suggested that a scheme for combining groups of unions might help to solve some of the difficulties, since, by removing the acute sick to a central infirmary, the objectionable practice—to which he referred as one of the curses of the Poor Law—of attendance on the sick by pauper inmates might be prevented. Mrs. Lele, of Leighton Buzzard, drew attention to the difference between the old *régime* and the new. Under the system of pauper nurses, she sa d. the patients died; now they lived, that was the difference. Other speakers, of whom several were ladies, advocated the continuance of the "useful inmate," whose services, they alleged, were greatly preferred by the senile and chronic cases. This valuable help, said Mrs. Ramsay, of Fareham, would not be available if the sick were removed to a central infirmary, and her view was supported by Sir

William Chance and others. Mrs. Shutt, of Salford, maintained that the numerous ophthalmic cases which came under infirmary treatment should have skilled attention; while Mrs. Pratt, Ashby-de la-Zouch, said that an entire staff of trained nurses was unnecessary. One guardian said that he thought niceties in nursing were being pushed rather too far, especially as the money came out of the ratepayers' pockets, and his remarks were received with applause.

### DENTAL NURSES.

The latest proposal in America is the creation of dental nurses. It emanates from a doctor, who, at the annual session of the American Medical Association, read a paper on the subject. The rather absurd suggestion is that nurses should be employed in dental work as well as in other branches of medicine and surgery. Their duties would largely consist in the enforcement of antisepsis, in the care and preparation of instruments, dressings, and operators' hands. His opinion was, however, that if this field of labour were thrown open to the trained nurses, it might become necessary for them to obtain special registration as trained dental nurses. Otherwise, he thought that there was a risk of nurses infringing the dental registration laws.

### IRISH HOSPITALS AND THE MIDWIVES ACT.

At the ordinary meeting last week of the council of the Royal College of Surgeons of England, the difficulty between the Irish training institutions and the Central Midwives Board respecting the certificates which must be produced before a candidate can be admitted to the board examinations, was mentioned in the statement read by Mr. J. Ward Cousins reporting the work of the Central Midwives Board for the past year. According to this statement, at a recent meeting of the board two conciliatory communications were read from the Irish hospitals, "but the board were unable to consider any alteration of the existing rules; and, therefore, it was probable that the matter would be referred for the decision of the Privy Council."

### THE ASYLUMS BOARD AND STATE REGISTRATION.

At the meeting of the Metropolitan Asylums Board on Saturday, the Hospitals Committee reported that they had considered a copy of a draft Bill for the State Registration of Trained Nurses, which had been presented to Parliament by a society which has been formed to promote that object, with a request that they might be furnished with any suggestions the Board might have to make. The committee thought that, having regard to the large number of nurses employed by the Board, steps should be taken to ensure the representation of the managers on the Council in the event of the Bill becoming law, and a recommendation to this effect was submitted. Miss Maud Stanley, commenting on the recommendation, said that the majority of the London hospitals were not in favour of the Bill, and that it would be a great injustice to the nursing profession if it were passed. Mr. J. G. Talbot having, however, pointed out that the recommendation would not commit the Board to any approval of the Bill, it was adopted. As we intimated last week, the introduction of the Bill means nothing. Any member can introduce a Bill into the House of Commons; the difficulty is to pass it, or even to secure a debate on the second reading. As a matter of form, Sir Frederick Dixon-Hartland has given notice of his intention to move the rejection of the State Registration Bill "when it comes on for second reading"; and the Associated Hospital Council, representing most of the principal London hospitals, has circularised members of Parliament in opposition to the measure. But next year, when Dr. Farquharson may have better luck in balloting, is the earliest time the Bill is likely to be discussed.

### THE NURSES OF THE ROYAL DEVON HOSPITAL.

In the report of the committee, treasurer, and medical officers of the Royal Devon and Exeter Hospital, to be presented at the annual meeting this week, the results of the examination of the nurses, by Dr. Acland, of St. Thomas's Hospital, are given. Dr. Acland states that of the candidates, two, Nos. 19 and 32, are to be specially commended for the excellence of their work, and the admirable manner in which they did both their papers and the *viva voce* examination. One, 28, he adds, showed in *viva voce* that her answers on paper did not adequately represent her value as a nurse. Dr. Acland was specially pleased with "the evidently superior education shown by the papers of all the candidates," and he expressed his thanks to the matron for the able arrangements she made by means of which the *viva voce* examination was greatly facilitated.

### NEW MATRON OF LEYDSDORP HOSPITAL.

We understand that Miss M. A. Borthwick has been appointed matron of the Government Hospital, Leydsdorp, Transvaal. She was trained at the Royal Hants County Hospital, Winchester. As a member of the Army Nursing Service Reserve, she served in South Africa for nearly three years, first on the staff of No. 11 General Hospital, Kimberley, and afterwards as acting superintendent at the Military Hospital, Vryburg, Bechuanaland, and at No. 16 Stationary Hospital, Mafeking.

### THE PROBLEM AT OLDHAM.

It was announced at the annual meeting of the Oldham Nursing Association that the debt to the bank at the end of 1903 was £350, an increase of £116 on the amount due at the end of 1902, that the fees for supplying nurses owing to the association at the end of 1903 amounted to £161 as against £72 at the end of 1902, the net result being that the organisation is in a worse position to the extent of £144 on the year's working. The problem of the managers is how to improve the position. The Chairman, in moving the adoption of the report, reproached the people of Oldham for only supporting the association to the extent of £108, and the amount is certainly very small. It would probably be much larger if the impression did not prevail in the town that the fees paid to the nurses cover the expenses of both departments. But as the Association has the two departments, and supplies nurses free to the sick, to persons who cannot pay a fee, the managers ought not to appeal in vain for the financial help they require to relieve them from anxiety about the future. We advise them to see that the sum represented by "fees owing for supplying nurses" is speedily reduced. The question of financial management, in fact, wants thoroughly overhauling.

### PROGRESS AT HUDDERSFIELD UNION INFIRMARY.

We are glad to learn that the Huddersfield Guardians have decided to arrange with the workhouse medical officer to give a course of lectures to the nurses and probationers in their service. For the course of lectures and for other work in connection with the training of the probationers the guardians will pay the medical officer a fee of £20; they have also resolved to expend £25 on the purchase of the books and apparatus necessary for the lectures.

### A NURSE KILLED WHILE CYCLING.

Last week an inquest was held on the body of Miss Alice Maud Stevenson, a nurse at the Sunderland Borough Asylum, who died from the effects of injuries due to an accident while she was cycling along one of the principal streets in the town. It appears from the evidence that Miss Stevenson, in trying to avoid a van standing in the road, ran in front of a tramcar. The car struck the front wheel of the bicycle, and the unfortunate young woman fell head foremost against the buffer of the tram. In summing up, the Coroner said that no blame could be attached to anyone. But it transpired that the deceased was a nervous rider, and that when she was leaving the Asylum on the morning of the accident she expressed some fear as to how she would get through the traffic of the town. In these circumstances, she should have been strongly advised not to attempt the task. Nervous cyclists run the most serious risks in thoroughfares which are crowded with vehicular traffic.

### "A HARD CASE."

Under the heading of "A Hard Case" one of the daily papers gives a report of an action brought by the Nurses' Co-operation, 8 New Cavendish Street, against a lady at Carshalton for £3 4s. The defence was that the nurse's patient was the young valet of a naval officer who had lodged with the lady and left her in debt. If so, he is a shabby person, but we presume that he can be brought to book. It is, however, beyond doubt that the services of the nurse were requisitioned by the lady of the house, and if it be a hard case that she should therefore have to pay the money, it would have been a harder one if the Nurses' Co-operation had been deprived of the fee which was admittedly earned.

### RESIGNATION OF NURSES AT AN IRISH INFIRMARY.

At the last meeting of the Cork Board of Guardians, a letter was read from three trained night nurses of the District Hospital tendering the necessary month's notice of their intention to resign their offices. The Visiting Committee advised that the resignations should be accepted, and that the nurses who had resigned be granted testimonials in respect of the very satisfactory manner in which they had performed their duties. No explanation of their action was given, and it was decided to advertise for one trained nurse at a salary of £35, rising to £45, and for "two trained, or qualified nurses," at a salary of £25, rising to £35. Is the "qualified" nurse, then, to make her appearance under the auspices of the Irish Local Government Board?

### THE UNCERTIFICATED MIDWIFE AT WORK.

At an inquest held last week in Macclesfield on a child who died two hours after birth, the coroner said that the uncertified midwife who attended the case of confinement "had sadly mistaken her duty and her calling." Instead of remaining with the child, which was born in a weakly condition, or fetching a doctor, she went away in an hour to attend another case, "not knowing that it was her duty to see that every effort was made for the child's life to be saved." He rejoiced to know that the Midwives Act would come into force very shortly, and that each midwife would be bound to have a certificate before she could attend a case. At present cases were sadly too numerous in which midwives did not seem to know their responsibility.

### THE NURSING STAFF AT THE RINGWORM SCHOOLS.

At a meeting of the Metropolitan Asylums Board on Saturday, the Children's Committee reported that they had had under consideration a letter from the dermatologist calling attention to the trouble and inconvenience occasioned by the frequent changes in the nursing staff at Bridge School, and suggesting that some inducement should be offered to the nurses to remain in the way of increased wages. During the last three years eighteen of the staff at the Bridge School had resigned, while at the Downs School no fewer than twenty-two resignations had taken place since February of last year. Dr. Fox, the dermatologist, in his letter stated that "at the present moment I have hardly a soul who is of any use, except the sister, and if she changes I have to begin my work of training all over again." In order to induce the sister or senior charge nurse to remain at the schools, the committee recommended that the senior charge nurse at Bridge School be designated "sister" and paid a salary at the rate of £40 per annum, rising by annual increments of £1 to £44 per annum, and that she be allowed the uniform of an assistant matron. With regard to the remainder of the nursing staff the committee while sympathising with Dr. Fox's desire to avoid frequent changes and to retain the services of nurses who had learnt the treatment, felt that the salaries now offered, from £20 to £22, were good, and that all they could do was to make those paid at the Bridge School uniform with those paid at the Downs School.

### SHORT ITEMS.

The St. Olave's Guardians have decided to establish a sick cookery class at their infirmary for the instruction of nurses in their third year of training, the instruction to be given at a time the nurses are off duty.—The annual general meeting of the Hammersmith and Fulham District Nursing Association will take place on the evening of Tuesday, March 15, at the Town Hall, Fulham, and among the speakers will be Mrs. Creighton and Lord Lytton.—The marriage of Miss Kate E. Bramwell, for nearly two years district nurse for the parish of St. Andrew's, New Kent Road, took place recently, the bridegroom being Mr. W. J. Sims, of Acton. The presents included many from the doctors and patients among whom the bride had worked.

### The Nursing Outlook.

"From magnanimity, all fear above;
From nobler recompense, above applause,
Which owes to man's short outlook all its charm.'

### OPEN-AIR TREATMENT.

MISS NUTTING, of the Johns Hopkins Hospital, which has lately had such a lucky escape in the great Baltimore fire, had a most interesting exhibit at the Tuberculosis Exposition in that city. Miss Nutting is a woman of thought as well as of deed, and has for many years been an advocate of treating tuberculosis patients at home if possible. From current advertisements on behalf of various Boards of Guardians for places where consumptive patients can be boarded out, it is evident that the plan is spreading in this country. The Guardians require houses where the patients will have proper care and attention. Now in order to secure this we venture to press on these Guardians and others that it is necessary to have such cases, if they are treated at home or boarded out, under the daily inspection and direction of a visiting nurse. Then the new methods of treatment can be carried on without the expensive special establishments, and the unnecessary herding together of tubercular cases. The district nurse everywhere has many consumptives under her charge at one time or another; but, unfortunately, with the rapid modifications of treatment, and the absence of post-graduate instruction, it is seldom that the district nurse has the knowledge or the capability to direct these cases aright. There are hundreds and thousands of consumptives in England who cannot enter a "sanatorium," who must sometimes go on working to the last and then go to the infirmary to die; their symptoms might be greatly mitigated, or in the early stage the disease might be arrested, by the daily oversight of a specially trained nurse. It is to be hoped that the reports and photographs which formed Miss Nutting's exhibit will be available at some English congress on nursing or health, and then the value of this system will be fully demonstrated.

For what are the leading characteristics of the new treatment which is proving so effective against one of our most fatal complaints? They are three:—(1) Fresh air and sunlight; (2) Proper feeding; (3) Graduated exercise. There is a fourth and very strong element which may well be called "moral influence," and it is one which the house-to-house visiting nurse must not forget. It is that part of the treatment which can best be carried on at home, for it includes fighting against the egotism of the disease, which was referred to in the medical part of THE HOSPITAL a fortnight since, and training the friends in the attitude to be adopted towards the patient quite as much as in training them how to avoid infection.

Now in many houses, though the family are not well off, it is possible to give the patient a sleeping room to himself and to make this room hygienic. Of course the first thing to do is to choose a room with as much light and air as possible—preferably with a southern aspect. In the suburbs, in some of the houses of our provincial towns and in most of our villages, it is possible to get a cheap shelter put up in the garden where the patient can sleep except in very bad weather. But if it is a room that has to be arranged, it needs windows that will open wide, no curtains, and as few stuffy things and useless ornaments as possible. It must not be forgotten that light is a germicide, and that it is not only fresh air one wants to get into the room, but also sunlight. Then the meals and their cooking and the seeing that they are eaten can all be taught once, and then supervised by the nurse. The actual dietary will, of course, be drawn up by the medical man, and it is advisable that the nurse should weekly submit to him a written report on each patient, giving details—such as increase or decrease of weight, amount of food, sleep, etc. Then the exercise, which must be regulated by the patient's condition, and which must be firstly under the physician's order, will also fall daily under the nurse's inspection. Of course where work has to go on outside the house the nurse has not so much control. It means so much that the exercise should be regular, should be taken in the most open district available, should not be hurried or curtailed for trifling interferences and regarded as of no importance. It is also necessary to see that the patient is properly clothed for his exercise, not laden with heavy coats or with his mouth covered by some insanitary wrapper. The question of clothing both by day and night is of the utmost importance, for we are all aware of the heavy outbursts of perspiration in these cases, which are so distressing and discomforting to the patients. The nurse can see that all clothing aims at lightness and warmth; she can teach how a quick sponge and change of nightgear between blankets can give the patient ease after a "night sweat" and send him off to sleep again. She can teach the family all the little devices which are known in hospital, and which can be carried out at home with the added charm of being amongst friends and out of the atmosphere of continual sickness which makes a ward depressing at times. With the means of preventing infection the nurse is sure to be acquainted, but she must keep up with the times and know all about paper handkerchiefs and other devices, and she must pass on her knowledge to the family. And she must sometimes visit at unexpected hours. Is it not evident that it is worth while to train all district nurses on these lines, or else to have special nurses to undertake the visitation of these very numerous cases?

### Lectures Upon the Nursing of Mental Diseases.

By ROBERT JONES, M.D.Lond., B.S., F.R.C.S.Eng., M.R.C.P.Lond., Resident Physician and Superintendent of the London County Asylum, Claybury.

### LECTURE VI.

OXYGEN is a necessity of life. It must be supplied to protoplasm in order that the energy we call life may continue. Oxygen is supplied to the tissues through the circulation of the blood and by means of the oxygen-carriers in the blood, viz., the red corpuscles. The blood not only carries oxygen and food to the cells of the different tissues, but it also removes the waste products of cell life, such as carbon dioxide, urea, and other salts.

*Respiration.*—The act of respiration is very distinctly under the control of the nervous system, more especially that portion of it called the medulla oblongata, in which lies the respiratory centre, close in situation to the vaso-motor and the cardio-inhibitory centre. The word "centre" merely means a special collection of nerve cells with their fibres, situated in any portion of the nervous system and governing certain definite functions. When the respiratory centre is destroyed respiration ceases. This centre acts in a rhythmic manner. It is excited by afferent stimuli from the lungs through the vagus nerves—the chief stimulus being the expansion of the lungs in inspiration and their collapse in expiration, and efferent messages are taken down the phrenic nerves (which supply the diaphragm) and the inter-costal nerves supplying other muscles. Respiration can be quickened directly by variations of the blood supply to the respiratory centre. If this blood supply be deficient respiration is more frequent and deeper. Respiration becomes laboured when the pressure of the air is about four times that of the atmosphere, and also when it is reduced to about one-third that of the atmosphere; also, when the oxygen falls to about 7 per cent. in place of the 21 per cent. in the atmosphere, or when the carbonic acid limit reaches 15 to 20 per cent., even though the oxygen is plentiful. The closeness of crowded rooms is due to an accumulation of carbon dioxide, as also to the diminution of oxygen, hence the necessity of free ventilation and free access of light and air, to which we shall refer later. The act of respiration consists in inspiration and expiration, and the normal rate is about 15 to 18 times a minute, although this rate is subject to many fluctuations, such as caused by the time of day, season of the year, the weather, occupation, the state of the emotions, attention, and muscular effort—all of which influence the respiration, either accelerating or retarding it. Each time that air is drawn into the lungs about one pint enters, and an equal amount is expired. This is called *tidal* air. With the deepest forced inspiration probably about three pints more enter. This is called *complemental* air. After ordinary expiration, which expels the tidal air, a certain quantity remains in the lungs, which may be expelled by a forcible and deep expiration. This is called the *reserve* or *supplemental* air. When forced expiration takes place after the deepest possible inspiration about eight pints of air are breathed, viz., the combined tidal, complemental, and supplemental air. The total quantity of this air is called the *vital capacity* of the chest, and machines are often seen at shows and fairs which register this. After the deepest expiration some amount is still left in the lungs, this is the *residual* air. During natural, quiet respiration there remain about five pints of impure air within the chest. This mingles, by diffusion, with one pint of pure air, inspired into the lungs, to which we have already referred. Thus we see that only a small proportion of the air within the lungs is changed at each complete act of respiration. The secretion from the air passages is just sufficient to keep the mucus surface moist. The bronchial tubes, the larynx, and nasal cavities are all lined with wavy moving cells so as to get rid of the secretion, and in certain states of ill-health the quantity and disposal of this secretion—which when in excess is called *expectoration*—may become a serious matter. At each expiration a certain quantity of carbon dioxide and water are got rid of, and it is also warmer, which makes the difference between inspired and expired air. The result of respiration upon the body is that arterial blood, by the aid of hæmoglobin in the red blood corpuscles, is able to take up oxygen, with which it parts through the lymph to the protoplasm of the tissue cells, which is always most ready to combine chemically with the oxygen. The reduced hæmoglobin takes up fresh oxygen from the air and again becomes oxy-hæmoglobin. This takes place in the air cells of the lungs, the oxygen of inspired air passing—by diffusion—through the walls of the air cells and the capillaries direct into the blood, and being thus picked up by the reduced hæmoglobin. The carbon dioxide formed and set free in the tissues passes from them by diffusion to the lymph, then through the walls of the capillaries and into the blood, where the sodium salts of the plasma readily combine with it. It reaches the lungs, where it is at a higher pressure in the blood than the carbonic acid of the inspired air in the lungs, and by osmosis passes through the capillary walls and diffuses with tne air contained in the lungs, being again expelled at each expiration. The total area of the air spaces in the two lungs, if laid out flat, would be larger than the area of the floor of a "single room"—about 100 square feet the floor area of a "single room" being 63 square feet. The chief seat of combustion and the formation of carbon dioxide is in the muscles. When at work the muscles take up more oxygen and give out more carbon dioxide than when at rest, and in cases of acute mania the respirations are often quickened, and fresh air and free ventilation become very necessary. When at rest less oxygen is used, and respiration in cases of melancholia or stuporose insanity becomes retarded. When respiration ceases, death occurs, for the prolonged absence of oxygen is incompatible with life.

*Digestion.*—The whole process of digestion from the mouth to the large intestine is under the control of the nervous system. The food is ground in the mouth by the teeth and there mixed with saliva from three pairs of glands, the sub-lingual, parotid, and submaxillary. The saliva owes its chemical action to the presence of an unorganised ferment or an *enzyme* called *ptyalin*, and its property or influence is *amylolytic*, that is, it converts the starchy food, such as bread, potatoes, rice, or grain, into soluble sugar, which is then absorbed. The time the food is kept in the mouth during mastication is not sufficiently long for the saliva to act chemically upon the starchy constituents. The ptyalin of the saliva can only act in an alkaline medium, so in the stomach the process of starch digestion is suspended, and it is only when the food gets from the stomach to the small intestine that the ptyalin begins to act again, and this it does in conjunction with the pancreatic secretion. Whilst in the stomach the food is acted upon by the *gastric juice* which also has its ferment or enzyme termed *pepsin.* This pepsin, at the body temperature, acts upon all proteids such as the white of egg, cheese, casein of milk, or meat foods, converting these into soluble *peptones*, which are then absorbed. The movements of the stomach are constant for three or four hours during the time digestion is going on, and these movements help to churn the food and to bring every part of it into relation or

LECTURES UPON THE NURSING OF MENTAL DISEASES—*Continued.*

connection with the gastric juice and its enzyme. After some hours the contents of the stomach—now called *chyme* —are discharged into the small intestine, there to mix with the *bile* secreted by the liver and the *pancreatic juice* secreted by the pancreas. The latter has two kinds of ferments, one amylolytic, like the ptyalin of saliva which together complete the process started in the mouth, and the other *proteolytic*, called *trypsin*, which is similar in action to the gastric juice with its pepsin. Trypsin acts upon the proteid matter, but it can only act in an alkaline medium, and the contents of the stomach are acid. To render these alkaline the pancreatic juice has a third quality, viz., its alkalinity, which, together with the same quality of the bile, enable the ferments named to act in an alkaline medium. It is owing to the alkalinity of the pancreatic juice and bile that both of these are enabled to act upon fats, which are emulsified or saponified. The food by this time is rendered into a state whereby the *lacteals* of the villi—the villi being very fine projections of mucous membrane within the small intestine, and which are composed mainly of blood-vessels and lymph-vessels—can take up the emulsified fats and pass them on through the lymph-vessels to the lymph system in the intestines and then into the *receptaculum chyli*, from which they discharge into the venous circulation. The *blood-vessels* of the intestine take up all the food which has been rendered soluble by means of the ferments, in contradistinction to the lacteals, which only take up saponified fats. The soluble food, such as the sugars and the peptones, which are taken up by the blood-vessels do not enter the circulation at once, but they are collected together into a large vessel, the *portal vein*, and then carried to the liver, where the portal vein once more breaks up into capillaries. The foods to some extent are stored up by the liver as *glycogen*, the liver allows the remainder to pass through into the lungs and into the general circulation. The glands of the small intestine, which are here only just referred to, also produce a secretion containing a ferment, which, however, is not proved to be of much importance in the process of digestion. In addition to these glands there are in the body a number of others called *ductless glands*—viz., the thyroid, the parathyroids, thymus, and suprarenals—which need not be studied by nurses, although they may have important functions. They are probably capable of marked reaction in the human organism, but little is at present definitely known concerning them.

### How Nurses are Manufactured in France.

Under the title of "A School for Nurses in 1903," a paper recently appeared in the *Revue des Deux Mondes*, which gives an extraordinary account of the way in which nurses are "manufactured," rather than trained, in France at the present time. The author, who claims to have gone through the whole course herself, appears to be a strong partisan of the old arrangement by which the management of the Paris hospitals was in the hands of religious communities; and perhaps for this reason some of her statements should be taken with a few grains of salt; but it is abundantly evident that the whole system is as different as can be imagined from the well-ordered methods of an English three years' training.

### One Year's Training.

To begin with, the training is for one year, in the case of the author of the article from October to July. The pupils are resident and non-resident, male and female, an arrangement that would not appeal to many British matrons. The outside pupils attend lectures and demonstrations in classrooms and in wards in three different hospitals, by no means very close to each other. The pupils are given a manual to study which begins, not as one might expect with an outline of the course to be followed, but with a pronouncement on clericalism, as if the handbook were intended to be a political treatise. After a day or two spent in the study of this work, the pupil has to be present at a practical demonstration of minor medicine. The account of the demonstration is rather comical.

### The First Lecture.

"I followed some nurses who appeared to have the same destination as myself. We entered a small room, furnished with a table and a few chairs. The windows were closed, and the atmosphere was stifling. Seated behind the table was the superintendent who was to give the course, waiting till the pupils should have assembled in sufficient numbers. Meanwhile I watched the arrivals. There were the nurses, ten or twelve in number, in cap and blouse, and seven or eight unattached pupils. These, like myself, came from outside. Twenty minutes passed. The lecture began. 'Ladies, we will speak to-day of the different plasters and ointments in ordinary use.' Then a voice interrupted: 'Mademoiselle, may I go away? I have two operations?' and without waiting for a reply, a little nurse went out, followed by two or three others, talking and laughing. The lecturer called one back: 'Elise, try to come on Wednesday.' But the reply came in a sulky voice: 'Wednesday? I can't, it is Adèle's turn.' And then the lesson went on, interesting and practical, given by a capable woman. Mademoiselle J. had left the profession of teaching to enter hospital. This is a very rare occurrence. Now she is a superintendent of the first class, to which fact her cap of black tulle, with silk strings, bears witness. The medicaments mentioned are shown us in turn, with a clear explanation, understood by everyone, of their properties and uses. The teaching was certainly good, and ought to bear fruit. But why do not the nurses attend it more regularly?"

### The Nurse of Many Duties.

Then follows an account of a lecture given in the old chapel of one of the hospitals on the various duties of the nurse, male and female, towards self, patients, colleagues, superiors, etc. While the professor, this time a man, holds forth on the lines of the manual, the nurses listen to his promises of the time soon coming when it will no longer be necessary for the same nurse who, early in the morning, has "performed the most repugnant tasks," and has afterwards washed the floors and the dressing-tables, to help the surgeon in the most careful details, and often, in default of other assistance, dress with her own hands wounds for which the most rigorous asepsis is necessary! But while the nurse is dreaming over these prophecies, the professor is continuing an enumeration of the superiorities of the lay nurse over her "religious" predecessor. He says, "How disinterested is the former! she is not working in hope of attaining Paradise! she is not attracted from the care of the sick by the need of saying her office!" and he comes to an end by assuring his audience that if they follow his advice they will obtain their diploma and the promotion to which they aspire. As far as money goes, their ambitions need not be very high. The assistant nurses begin with 30 francs a month, rising to 37 francs, or about £18 10s. a year. But a rise was promised for 1903, of what dazzling amount was not mentioned.

### HOW NURSES ARE MANUFACTURED IN FRANCE—*Continued.*

### Payment from Patients.

Later a very serious indictment is brought against the system which allows the nurses to levy small sums from the patients in return for necessary services rendered to them. One or two sous, or even more, are paid for indispensable care. And as these patients are generally drawn from the same poor classes as our hospital patients, it is not nice to think of the hardships that might be inflicted by these means on the patient as well as on his family who have to provide the money.

### Death-Traps for Dormitories.

The nurses are as little considered as their patients, so far as their comfort goes. The words of a French physician are quoted, who says: "I know dormitories which are veritable death traps: through the tiny, ill-fitting window, the rain comes by day, and the cold by night." In one hospital they are not much better than rat holes, hidden away under the roof of the oldest part of the building. Dirt and disorder reign supreme. "In these garrets, the beds are so close together that the feet of one nurse touch the pillow of the next. Worse details I pass by."

### Nurses Sleep through Lectures.

Another lecturer gives a course of instruction meant chiefly for those who wish to become private nurses. The manual announces that the chapter on this work will be supplemented by a living demonstration. It is certain that this might be of much use, if properly carried out. But almost before the lecturer has announced that he is about to begin, the little nurses have crossed their arms and settled down for a nap. He profits by their action to mention that they may sleep if they like, but that they are not to make a noise. However, the reprimand does not hinder that part of the audience which is not sleeping from talking, laughing, and passing round caricatures in the worst possible taste. The professor abounds in anecdotes, without ever coming to the actual subject of his lecture; and at the end of the hour the nurses have not added one useful item to their stock of knowledge.

### A Useless Examination.

A month after entrance an examination is held, which is scarcely anything but a mockery. All the pupils present themselves armed with the inevitable manual, and proceed to copy the answers to the questions as seem best to them. Suddenly the superintendent, perhaps in a worse temper than usual, makes a raid on all the books, and everyone's pen comes to a stand and the pages remain blank! The greater part of the outside pupils are working for their diploma with the idea of becoming either "lady delegate of the public service" or lady visitors of the dispensaries. The diploma is the only necessary qualification, but the posts are given by private influence, often political.

### Surgical Work in the Wards.

In February the nurse began her course in the women's surgical ward of the "Hospital de la Pitié." She speaks with admiration of the exquisite cleanliness of everything, and of the motherly superintendent moving up and down her ward with a kindly smile, evidently loved by her patients. There were 40 beds, tended by a head assistant and two juniors. "The head surgeon," the author says, "arrives late in the morning. Before then a dresser goes his round, being helped by one of the juniors. And the serious inconvenience is at once apparent, especially in a surgical ward, of the want of division of the work among the attendants. The nurses, careful as they may be, cannot hide the traces of the rough work which has occupied them since five o'clock in the morning. How can service of this kind be compatible with the minute cleanliness exacted by careful asepsis? And what an overwhelming task to prepare the dressings and all the thousand indispensable accessories! The day is hard for these girls. Also they all take their week's turn of night duty. So that I can easily understand the irresistible drowsiness which overtakes them in those evening lectures, too uninteresting in themselves to keep their poor exhausted brains awake."

### The Lay Figure Josephine.

The lay figure, nicknamed "Josephine" plays a good part in the year's work. At one lecture she was missing. It was a bandaging class, and in "Josephine's" absence the professor called up one of the nurses to take her place on the table for him to give his demonstration. The class, it should be remembered, was a mixed one. The lesson lasted an hour. The author brings two accusations against the hospital, the first that the staff is not nearly equal, even in number, to the needs of the patients, and the second and more serious charge that in the wards every young girl, whether patient or nurse, loses her sense of modesty. It would be ridiculous, so the general feeling goes, to be shocked at any want of decency. And the fear of ridicule is stronger than any other authority. She speaks of the care in great operations, requiring time and expense, which are freely lavished upon them; but the ordinary, every day cases, she says, are put off from day to day till, perhaps, it is too late to do anything for their relief.

### A Smattering of Many Subjects.

As the year went on the work became more fatiguing by reason of the number of subjects of which the pupils had to obtain a smattering. Minor surgery and medicine, care of babies and women, hydropathics, vaccination, massage, all found their place in the day's course. The account of the teaching of treatment by douches is particularly revolting, the victims being idiot boys from one of the asylums, who were stripped while each nurse was shown how to use the jet on their bare skins.

### Final Examination.

The year ends with a public examination. The candidate first passes before the judges to answer questions as to her name, age, place of birth and profession. This being answered the candidate goes before the head midwife, who judges her capability for dressing a baby. The necessary materials are given the candidate, and upon a cardboard baby, already familiar to the probationer, she exercises her maternal instincts. Next, before the chief, in front of whom "Josephine" is reclining upon a long table, the candidate mounts the platform, and by his order places a double spica on her shoulders. Then she puts together an aspirating apparatus, recognises some medicines, naming the dose, and the chief uses of each, and the examination is over.

### Prize Day.

A week later is prize day. The author of the article had obtained maximum marks in all but one subject, of which she was told that it would not do for her to be at the head in every subject! So her ten marks were reduced to nine and a half. The prizes were books, and those selected were the "Letters of Madame de Sévigné" and "Lost in the Ice," illustrated, and both gorgeously bound in red and gold!

The previous year all the prizes had been copies of a well-known anti-clerical treatise.

### Everybody's Opinion.

[Correspondence on all subjects is invited, but we cannot in any way be responsible for the opinions expressed by our correspondents. No communication can be entertained if the name and address of the correspondent are not given as a guarantee of good faith, but not necessarily for publication. All correspondents should write on one side of the paper only.]

### THE LAST EXAMINATION PAPER.

"R. J. R." writes: "Belinda," in her examination paper on Abdominal Fomentation, says she "would put the jaconet wool and binder to the fire." I presume she puts them there to warm. I think it very unnecessary to put the jaconet and wool to warm, and a very dangerous thing to put wool near the fire. "Nurse Betty" speaks of sprinkling turpentine or liniments on the flannel. I was always taught that when turpentine or liniment is sprinkled on the lint or flannel to call it a stupe, and not a fomentation.

### DISTRICT TRAINING AT KEIGHLEY HOSPITAL.

"AN INFIRMARY MATRON" writes: I was glad to read in last week's issue that during a probationer's training some district nursing or private work is accomplished at Keighley Hospital. I may say that in our infirmary in Lancashire some years ago the plan was discussed and advocated; but two obstacles could not be overcome. One was the housing of the extra nurses, and the other was the distance the nurses would have to cover. The infirmary was situated well out on one side of the town, and it was reckoned that a nurse would often have to go seven miles to visit some of the poorest parts. I for one shall be glad to learn how they manage at Keighley. The longer we live in institutions, I feel, the more fully we are convinced that the three years spent in a hospital to gain a certificate leave much to be desired in the average nurse, and the more varied her experiences can be made, the better the result. Sir William Bennett has spoken admirably to nurses. He has put before them what a doctor wants in private practice—namely, a nurse who knows both her work and her place. The criticisms, I think, no sensible woman will object to.

### SIR WILLIAM BENNETT ON PRIVATE NURSING.

"MEDICO" writes: Most of the correspondents whose letters have appeared in THE HOSPITAL on the subject of Sir William Bennett's remarks on the removal of the words "decency" and "indecency" from a nurse's dictionary appear to have opinions decidedly opposed to what he stated. It seems to me rather odd that all these correspondents should refer to dressings done in the wards, and operations performed and dressings put on in the theatre are not mentioned once. How is it that in the theatre nurses are handing sponges, dressings, etc., to the operators while there is the greatest exposure, no account whatever being taken of this, either by the nurses themselves or the surgeons (or your correspondents), and yet as soon as the same patient is back in the wards again, behold, the very same nurses it may be, will object to helping dress him on account of the exposure produced? Surely, if the patient has no objection to a nurse helping to dress him, the nurse to be consistent should have none either. It is the patient's business to complain, and not the nurse's. Either she must refuse to be present at operations which she would describe as "indelicate," and demand male nurses at those operations, or else she should raise no objection to assisting at the ward-dressing if required.

### Where to Go.

HIS MAJESTY'S THEATRE, MARCH 1.—Matinée performance, by distinguished artists, in aid of the National Hospital for the Paralysed and Epileptic.

ROYAL INSTITUTE GALLERIES, PICCADILLY, MARCH 1, 3 P.M.—Musical and dramatic matinee in aid of the Rural Midwives Association.

### Appointments.

ASHTON-UNDER-LYNE UNION INFIRMARY.—Miss Alice Radcliffe has been appointed night superintendent. She was trained at the Chorlton Union Hospitals, West Didsbury, Manchester, and has since been sister in the maternity ward at the Halifax Poor-law Hospital. She has also done private nursing. She holds the L.O.S. certificate.

BIRMINGHAM SKIN HOSPITAL.—Miss Rumble has been appointed matron. She was trained at the Bristol Royal Infirmary and Milton Fever Hospital, Portsmouth. She has since been sister in charge of the out-patient department in the Bristol Royal Infirmary.

BRADFORD UNION INFIRMARY.—Miss Ruth Broughton has been appointed ward sister. She was trained at Eastville Workhouse Infirmary, Bristol, and has since been nurse at Colchester Union Infirmary, staff nurse at Plaistow Maternity Charity, and manager of the Branch Midwifery Home at East Ham.

CARDIFF INFIRMARY.—Miss Margaret Phillips has been appointed ophthalmic sister. She was trained at Cardiff Infirmary.

MILTON HOSPITAL, PORTSMOUTH.—Miss Gertrude Mary Palmer has been appointed sister. She was trained at Fir Vale Infirmary, Sheffield, and has since been charge nurse at the South-Eastern Hospital, London, and sister at Fir Vale Infirmary, Sheffield.

ST. LEONARD'S INFIRMARY, SHOREDITCH.—Miss Annie Heaton has been appointed ward sister. She was trained at University College Hospital and St. John's House, London. She has since been lecturer to the London School Board, and sister at Plaistow Fever Hospital.

STRAND UNION WORKHOUSE, EDMONTON.—Miss Annie Tyers has been appointed superintendent nurse. She was trained at Southwark Union Infirmary, East Dulwich, and has since been night superintendent at the Central Sick Asylum, Hendon.

WALLSEND AND WILLINGTON QUAY JOINT HOSPITAL.—Miss Margaret Schwappe has been appointed nurse matron. She was trained at the Royal Infirmary, Newcastle-on-Tyne, where she has since been sister. She has also been matron of the private hospital West Hartlepool, and sister-in-charge of the Nurses' Home, Haworth.

### Novelties for Nurses.

BY OUR SHOPPING CORRESPONDENT.

### ROYAL ADMIRALTY SERGES.

FROM Mr. James Beattie, of Wolverhampton, I have just received some capital serge patterns, in navy, black, scarlet, and cream, which he assures me are proof against sun and storm. They are guaranteed fast dyed and shrunk, and woven only from the finest British yarns; and it is claimed that they do not spot with rain, nor change their colour if exposed to sea-water, or even if boiled in hot water, soap, and soda. One could hardly require more of any dress material, even that of a nurse's uniform, which is frequently subjected to fairly severe tests, especially when worn in district work; and my readers would do well to send to this firm for patterns. The navy serge, which is, of course, most obviously suited for uniforms, is manufactured in a variety of qualities, ranging in price from 10¾d. to 3s. 6d. per yard, double width; one very nice diagonal being 49 inches wide. The price of this is 3s. 6d. per yard. There is an equally large choice in black, which, if nicely made up, is eminently suitable for "off duty" wear in the form of the indispensable coat and skirt; while the nurse whose duty takes her to the tropics will like to know that she can have a cool-looking white serge from 1s. 0¾d. per yard, the prices and qualities ranging from fine to coarse, and from the cost just mentioned to 1s. 11½d., all double width. Nurses should make a note of the address:—Mr. James Beattie, The Royal Admiralty Serge Warehouse, 73A to 78 Victoria Street, Wolverhampton.

### Echoes from the Outside World.

### The King and the Navy.

On Friday last the King went to Portsmouth. Arriving at the Dockyard at 1.42 attired in the uniform of Admiral of the Fleet, he was received by Admiral Sir John Fisher and the principal naval and marine authorities in the port Subsequently he drove to Admiralty House, where he had lunch. He then embarked in the Admiralty steam barge and inspected submarine "A. 1," afterwards going on board the *Thames* to see the periscope and various details of the submarine service. Having witnessed the submarines manœuvring round the *Thames*, he visited the *Victory* and saw the cockpit arranged with all the fittings as they were at the time of Nelson's death. His Majesty next inspected the Museum and other points of interest on board and was shown the system of working signals as practised in the signalling school on board the *Victory*. On Saturday he embarked on the Royal yacht *Alberta*, and as the vessel steamed through Spithead a programme of evolutions and manœuvres was carried out by flotillas and submarines. The King is understood to have been much pleased with and interested in this naval exhibition. On landing at East Cowes his Majesty drove to the engineering shops belonging to the Naval College, saw the cadets at work and at dinner. When he entered the dining-room they rose from their seats, and, on the call of one of their number, "The King" was drunk in ginger-beer, followed by a round of hearty cheering. On Sunday, after attending Divine service in the dock-yard church, his Majesty inspected the girls from the Orphan Home, and the marines, who were drawn up in the garden of Admiralty House. He then went on board the *Cumberland*, one of the latest armoured cruisers, and in the afternoon made a tour of the dockyard and of the naval barracks, concluding the day with a visit to Eastney Barracks and town.

On Monday morning the King visited the Naval Gunnery School at Whale Island. Having inspected the sailors and boys under training on the parade ground, and various appliances invented by Captain Scott, which are now in the progressive course of instruction, through which all the officers and men qualifying in gunnery are now passed, he proceeded to witness a demonstration of how a naval brigade work on shore. A wall had been built up to represent a fortified position, the naval skirmishers "rushed" the wall, and were followed by another contingent with a field gun. As the gun would not go over the wall, dynamite was used to blow up a portion of it large enough to admit the cannon. The King was much pleased and amused at the naval display. Afterwards, at Portsea Island, he saw demonstrated the various ways of using submarine mines and torpedoes. His Majesty lunched at Admiralty House, and then returned to town. In the afternoon the following signal was made by Admiral Sir John Fisher:—"His Majesty the King has been graciously pleased to signify his extreme satisfaction and gratification with all that his Majesty witnessed during his visit to Portsmouth."

### The Queen at Brighton.

On Saturday the Queen paid a private visit to Brighton for the purpose of spending an afternoon with Princess Louise, Duchess of Fife, on her birthday, travelling in a special saloon attached to the 11.40 ordinary train. The intended visit of her Majesty had been kept so secret at Brighton that her arrival at the Central Station was almost unobserved by the general public. But the news that the Queen was in the town spread rapidly, and a large crowd assembled at the station for the purpose of greeting her when she made her appearance for the return journey at a quarter to six. They were purposely misled by the officials as to the entrance she would come in by, so that she was on the platform before they were aware of it. However, a large number succeeded in seeing and cheering her before she left, and she smilingly bowed her acknowledgments.

### The King and Lord Roberts.

The King has issued, through the Army Council, an Army Order, expressing his deep regret at taking leave of Lord Roberts, on his retirement from active employment, after fifty years in the service of Queen Victoria and his present Majesty in India, in Africa, and at home, with the highest distinction. The King affirms that he cannot part with his Commander-in-Chief without returning publicly to him his thanks and those of his army for the invaluable services he has rendered to the Empire.

### The War in the Far East.

Before the Siberian Rifles departed for the Far East the Czar reviewed them, and reminding them that their foe was "brave, confident, and crafty," wished them success. An official *communique* has since been issued in St. Petersburg stating that much time was now necessary to strike at Japan blows worthy of the dignity and might of Russia. A hundred surgeons and nurses left Odessa on Sunday for the front. A St. Petersburg telegram says that three Japanese officers, disguised as coolies, who were arrested by the frontier guards, were attempting to blow up the railway bridge over the Sungari River, in Manchuria. They were brought before a Court-Martial, condemned to death, and hanged on the bridge. The report that 70 miles of railway track and some important bridges have been destroyed between Harbin and Vladivostock is confirmed.

### The New Premier of Capetown.

In consequence of the general election in Cape Colony resulting in the resignation of Sir Gordon Sprigg, Dr. Jameson has, at the request of the Governor, undertaken to form a Ministry. He himself is Premier and Minister of Native Affairs. Dr. Jameson was the author of the famous Raid a few years ago. He is now the leader of the Progressive party at the Cape, and it seems to be expected that he will prove a strong and capable head of the Government. His colleagues include Sir Lewis Mitchell, the highest authority in the colony on financial matters, and Colonel Crewe, who defeated the Bond leader, Mr. Sauer. The new Parliament will meet next week.

### The Twentieth Century League.

The Bishop of London presided at the annual meeting of the Twentieth Century League, which was held on Monday afternoon at London House. There was a large attendance, and the Bishop, in opening the proceedings, said that the League was originated three years ago for the purpose of trying to supply every boy and girl in London with something to do and somewhere to go in the evenings after their work. The League wanted to send every Metropolitan branch before the work of next winter begins, £100. For this a sum of £3,000 would be required, and he was glad to announce that the Princess of Wales had sent ten guineas that afternoon. Her Royal Highness was unable to come to the meeting, but she wished well to the movement. Sir Henry Burdett dwelt on the urgent need of men and women to carry on the work, and expressed his surprise that we as a Christian people should be content to sit at home, criticise and shudder, and yet do nothing when we heard of an outbreak of hooliganism.

### A Book and its Story.

### MR. HALLIWELL SUTCLIFFE'S NEW NOVEL.*

THERE is extraordinary strength and power in Mr. Halliwell Sutcliffe's novel "Through Sorrow's Gate," and its atmosphere is as healthy and stimulating as the air of the moorland on which the scene is laid. The masterly delineation of the characters, the wild setting of the scenery, and the refinement which dominates the whole, gives an added grace to the writing of Mr. Sutcliffe, who is both a realist and impressionist—

"Pass sorrow's gate and far beyond
There's manhood waits you there."

These unsigned lines give the clue to the history of Griff Lomax the hero. Years before the story begins he had left the past behind him, and had come out on to the moors to live the life of a lonely recluse. By spending his days in hard wrestling with the moorland soil, he hoped to accomplish redemption for a past misdeed wrought in a moment of madness, the memory of which haunted him unceasingly. He comes on the scene in the first page. "The man stood at the door of his rude hut and watched the storm of snow begin. The first flakes came drifting down thinly, without purpose so it seemed, down the chill breeze from the north; they were not thick enough as yet to hide the desolation of the moor, which strode mile after hilly mile to the four corners of the sky. . . . This man lived alone upon the heath, had wrestled long with wind and rain and churlish soil. He seemed to be a part of the heath, a growth from the strong loins of the land that shut him in. It was not his height nor his breadth of shoulder that gave him kinship with the larger nature; it was a certain steadfastness which showed in every line of his clean-shaven face, a certain fixed hard set of jaw, a certain light, prophetic almost in its brilliance and wistful surety of faith, that shone in the clear hazel eyes." As he stood, looking across the moor, his thoughts wandered back to his boyhood and youth. To the far-off days when, as a Lomax and Lord of the Manor, he held a position among the landed aristocracy of the county. He was Lord of the Manor still, in spite of his voluntary exile, and the income from his lands was accumulating, with other moneys, from year to year. The spot he had chosen for his retirement was one around which strange stories of his wild ancestry lingered. Here in former times a Lomax, "tired of the world's emptiness," had set to work, as Griff was once more doing, to reclaim the waste moorland and had given the name of Withens to the intaken lands. "Intaking of the heath—the making of good land from the barren—had been a passion always with the moor folk. It had acquired a dignity, a measure of romance, a power to thrill the fancy and stiffen each man's fibres, which were possible only among a people whose far back fathers had felt the love of soil for soil's sake, of fight for fight's sake." For uncounted years the place had been named by the country-folk Lost Withens. Griff's ancestor had fought the fight with the moor and died in the midst of his labours, "and none had followed him to this world-end land." Strange, eerie stories had crept around the spot until "it became to be haunted by some of the myriad phantoms which any man might meet on his homeward way after nightfall."

In one of the many fine passages in which the book abounds, the author brings out forcibly the working of Griff's mind, as from the stormy scene without the hut, he turns inward to find, in the region of his own soul, a similarity between the circumstances of his life, and the storm before him. Griff had one faithful companion in his dog Trash, who shared all his moods and showed a sympathetic interest, so far as a dumb animal can, in everything that concerned them. "With the dog, and with his secret, Lomax dwelt aloof in this wind-driven spot. He seemed content; and always when he looked as now across the moor, he was truly constantly amid the greater silences. He had gone through the gates of sorrow once for all—those gates which each of us must pass if we would find the deeper pain and the livelier joy that await us in the shrouded lands beyond. Long, long ago he had left the gates behind him; long ago he had traversed the dark ways which test the greatness of a man to persevere and falter not; and now at last he was journeying slowly, groping as he went, towards the goal of recompense and heartease."

Griff had no neighbours, but he had friends among the scattered farm homesteads on the hills and in Marchcotes village, which was on his property. Gabriel Hirst the preacher was one. "A man worth looking at a second time was Gabriel Hirst. A first glance showed only a sturdy thick-set man of middle height with a rough hewn face and crisp black hair; but a second glance revealed a sort of hidden beauty, for he had travailed with his own soul and with others, had watched upon the hilltops for the messages which he afterwards delivered red-hot from the pulpit, had gathered strength and wrath and pity from his own experience, and had given out the same with an unsparing hand. . . . In all his words, in all his deeds, this man was honest as the sunlight and the storm; and Griff, though he had no care for his teaching, had loved him as a brother since his boyhood days." Gabriel was the one man who knew the secret that had driven Griff into the wilderness. He was one of the few visitors that came to the hut, and his visits were directed by an object in view, frequently a warning against some present or future danger, which was delivered in a form half mystic and half real, the outcome of his reflections upon the floating scraps of gossip that reached him, mingled with his own observations and much communing on the hillsides. These combined influences were generally brought to bear upon the person for whom the warning was intended in the form of a "vision" by which the preacher had been visited. Griff was, unknown to himself, on the eve of a new destiny when Gabriel Hirst called to warn him of a danger which he feared. But as this had to do with a woman who had no attraction for him, he was not moved in any way by his friend's rugged anxiety. He walked out to the door of his hut once more. To him "who had known passion, had understood those loves of women that have deepest roots of all—love of a strong mother for her only son, love of a strong wife for the strong man of her choice, had felt the hope that had turned liar when his heir was born and died," the "warnings" of Gabriel Hirst were as nothing. One more quotation, only: "Just as the storm had brought him disaster once, it was to come with fairer gifts now that he had trodden weakness under foot. Yet had he guessed that the ambassador would come in woman's gear, with eyes as deep as an autumn gloaming and a face that harboured weariness and pride this man of loneliness would have put a bolt to his hut door and sought safety on the other side. "Through Sorrows Gate" is a book that will live, and it is one of the few novels that every appreciative reader will wish not only to read, but to posesss.

* "Through Sorrow's Gate:" a Tale of the Wintry Heath. By Halliwell Sutcliffe. (Fisher Unwin. 6s.)

### Notes and Queries.

**FOR REGULATIONS SEE PAGE 277.**

*Birth Certificate.*

(180) With reference to the question of Nurse H. in our issue of February 13, the Secretary of the Obstetrical Society of London informs us that the official birth certificate is required and must be presented by candidates for examination.

*Army Nurse.*

(192) Will you kindly tell me how I can be trained for an army nurse? Must I go to a civil hospital first or do I go straight to a military hospital?—*En-Lora I.*

You must be trained in a civil hospital first.

*Maternity Nurse.*

(193) Is there any recognised rule as to the payment of an obstetrical nurse whilst waiting in a patient's house? Does she receive full fees, or only half fees?—*E. F.*

The nurse usually receives full fees from the date the case is booked for, unless otherwise agreed.

*Nurses for Inebriates.*

(194) Can you tell me if nurses are employed in homes for inebriates, and if so, how to obtain work in one? *M. A. I.*

Yes. You can only obtain employment in such homes by advertisement, or through friends.

*Home.*

(195) Will you kindly tell me of a home where a child could be placed without payment, as the parent wishes to give it up altogether?—*Nurse B.*

There is no home which relieves parents of their responsibility.

*Child's Nurse.*

(196) Where can a young lady get two or three months' cheap training? She wishes to be a lady nurse able to take baby after the first month.—*B. B.*

The Norland Institute, 10 Pembridge Square, W., the Liverpool Ladies' Sanitary Association, 8 Sandon Terrace, Liverpool, Miss Biddulph Pinchard, Court Royal, South Norwood Hill, S.E., all train ladies as children's nurses. Write and ask terms.

*Midwifery Training.*

(197) Will you kindly tell me the names of the cheapest lying-in hospitals in London for training in midwifery?—*No Name.*

Intending maternity nurses are advised to consult the Secretary of the Association for Promoting the Training and Supply of Midwives, 12 Buckingham Street, Strand, W.C. Amongst the cheapest schools are the British Lying-in Hospital, Endell Street, St. Giles, W.C., the City of London Lying-in Hospital, City Road, E.C., etc. See "The Nursing Profession: How and Where to Train" for full list, and for particulars of courses and terms.

*Stewardess.*

(198) Can you tell me:—1. The conditions under which stewardesses work? 2. What is the remuneration? 3. The age limit? 4. What are the special qualifications? and 5. Is such a post easily obtained?—*Inquirer.*

1. Conditions vary on different boats. 2. No fixed scale of remuneration. 3. No age limit. 4. Suitability. 5. Posts are very difficult to obtain.

*Hospital Training.*

(199) Will you kindly tell me how I can get a three years' certificate? Will a London hospital take me? I have had one year's training. If I went to the National Hospital for two years' training in massage would it disqualify me for general training afterwards, or would it be to my advantage?—*Training.*

Apply to matrons of suitable London hospitals, for which see "The Nursing Profession: How and Where to Train." You had better take massage after general training, not before.

**Standard Nursing Manuals.**

"The Nursing Profession: How and Where to Train." 2s. net; 2s. 4d. post free.

"Nursing: Its Theory and Practice." (Revised Edition). 3s. 6d. post free.

"Elementary Anatomy and Surgery for Nurses." By William McAdam Eccles, M.D.Lond., M.B. 2s. 6d. post free.

"Elementary Physiology for Nurses." By C. F. Marshall, M.D., B.Sc., F.R.C.S. 2s. post free.

"Ophthalmic Nursing." By Sydney Stephenson, M.B., F.R.C.S., 3s. 6d. net; 3s. 10d. post free.

"Nursing in Diseases of the Throat, Nose, and Ear." By P. Macleod Yearsley, F.R.C.S.Eng., M.R.C.S. 2s. 6d. post free.

"Fevers and Infectious Diseases." 1s. post free.

### For Reading to the Sick.

### SOWING AND REAPING.

Sow ye beside all waters
Where the dew of heaven may fall,
Ye shall reap if ye be not weary,
For the Spirit breathes o'er all.
Sow though thorns may wound thee;
One wore the thorns for thee;
And though the cold world scorn thee,
Patient and hopeful be.
Sow ye beside all waters
With a blessing and a prayer,
Name Him whose hand upholds thee
And sow thou everywhere.

Sow when the sunlight sheddeth
Its warm and cheering ray,
For the rain of heaven descendeth
When the sunbeams pass away.
Sow when the tempest lowers,
For calmer days will break,
And the seed in darkness nourished,
A goodly plant may make.
Sow when the morning breaketh,
In beauty o'er the land;
And when the evening falleth,
Withhold thou not thy hand.

*Anna Shipton.*

The little grain of wheat—a tiny, insignificant object—falls into the cold ground and for weeks is forgotten; it dies, it is transformed in some way, and then appears the blade, then the ear, then five or six goodly blades, with full corn in the ear, all sprung from that small beginning: and, if these be sown again, they multiply afresh, and soon you have a field of gold and waving corn. What a harvest from such slight material! And every Christian soul can produce the same. There are in every one immense possibilities of good: given the grace of God and good-will, we can all reach sanctity, as indeed we must do ere we see God. But no one ever was able to do great things for God "unless he fall into the ground and die," by humbling himself before God, accepting his lot with joy and gratitude, looking to God for everything, and ever striving to love God's will alone and most perfectly to fulfil it.

I thirst, my Master, to be at work for Thee, producing fruit in Thy vineyard. But I am sick and weak; I seem equal to so little; can I ever hope "to bring forth much fruit?"

Let me ask one more question, dear Lord, and then I will rest content. Will there be a harvest of souls somewhere gathered at some time, and will my pains have any share in producing it? O yes, my child, if the grain of wheat fall into the ground and die, it will produce much fruit. Let your life be hidden with Christ in God; not a prayer, not a twinge of pain, not a moment of desolation is lost in the life of a soul that rests humbly at the foot of the cross, that dies to self, that seeks not its own glory but that of its Maker, whose meet it is to do the will of him that sent it into the world.—*Anon.*

